# PARKINSON’S DISEASE

**DOI:** 10.1093/geroni/igz038.2127

**Published:** 2019-11-08

**Authors:** Madeleine Hackney

**Affiliations:** Emory University, Atlanta, Georgia, United States

## Abstract

The risk of Parkinson’s Disease (PD) increases with age as over 90% of individuals are diagnosed after the age of 50. Older individuals with PD face a compounding burden of reduced muscle power, decreased muscular endurance, and weakness related to disease specific processes such as an altered pattern of motor unit activation, rigidity and bradykinesia. Individuals with PD demonstrate a tendency towards type I fiber hypertrophy, and a greater heterogeneity for type II fibers. Despite the disease specific burden of PD, exercise results in improved mobility, balance, and movement initiation. Interventions like resistance training have resulted in strength gains, and other interventions, such as tango dancing may be particularly useful for balance improvements, as the movement involves multi-directional perturbations and whole-body coordination. Here, we will discuss the use of novel exercise interventions, such as resistance training and tango, to improve the muscle and mobility function of older adults with PD.

